# Long Term Depression in Rat Hippocampus and the Effect of Ethanol during Fetal Life

**DOI:** 10.3390/brainsci7120157

**Published:** 2017-11-28

**Authors:** Olivier Pierrefiche

**Affiliations:** INSERM ERI-24, GRAP, Groupe de Recherche sur l’Alcool et les Pharmacodépendances, Centre Universitaire de Recherche en Sante CHU-Sud, Université Picardie Jules Verne, Chemin du Thil, 80025 Amiens, France; op-lnc@u-picardie.fr; Tel.:+33-0-3-2282-7932

**Keywords:** ethanol, long-term potentiation, long term depression, hippocampus, memory, prenatal, fetal, GluN2B, KCC2

## Abstract

Alcohol (ethanol) disturbs cognitive functions including learning and memory in humans, non-human primates, and laboratory animals such as rodents. As studied in animals, cellular mechanisms for learning and memory include bidirectional synaptic plasticity, long-term potentiation (LTP), and long-term depression (LTD), primarily in the hippocampus. Most of the research in the field of alcohol has analyzed the effects of ethanol on LTP; however, with recent advances in the understanding of the physiological role of LTD in learning and memory, some authors have examined the effects of ethanol exposure on this particular signal. In the present review, I will focus on hippocampal LTD recorded in rodents and the effects of fetal alcohol exposure on this signal. A synthesis of the findings indicates that prenatal ethanol exposure disturbs LTD concurrently with LTP in offspring and that both glutamatergic and γ-aminobutyric acid (GABA) neurotransmissions are altered and contribute to LTD disturbances. Although the ultimate mode of action of ethanol on these two transmitter systems is not yet clear, novel suggestions have recently appeared in the literature.

## 1. Introduction

Alcohol in beverages is the chemical molecule ethanol, which can be considered a “dirty drug” since it has no specific molecular target on cell membrane to induce its effects on organisms. Ethanol is a small molecule, composed of two carbon atoms with a hydroxyl group. This gives it both hydrophobic and hydrophylic properties, allowing it to pass easily through most cellular membranes in the body. Thus, ethanol has no biological barrier and, when it is consumed by pregnant women, crosses the placental barrier to reach the fetus. At this stage of development, however, the fetus and the embryo do not have mature ethanol metabolizing enzymes (i.e., alcohol dehydrogenase (ADH)). Therefore, the action of alcohol lasts much longer in the fetus than in the mother’s body [1]. The effects of alcohol during pregnancy are multiple and largely irreversible because ethanol is a powerful teratogenic substance, strongly interfering with the central nervous system (CNS) developmental program. Indeed, ethanol may be the most dangerous drug for the fetus and is the most teratogenic [2]. Ethanol is pharmacologically classified as a psychoactive substance with a main depressive action on the CNS. It also has rewarding effects via its action through the brain reward circuit, which may explain the development of alcohol dependence. In the CNS, ethanol has a large repertoire of actions resulting in psychomotor depression, a reduction in motor coordination, logical reasoning deficits, and difficulties forming new memory and storing information. Despite our knowledge of the deleterious effects of alcohol as a drug of abuse and as a teratogenic chemical, recent surveys in Western countries revealed that about 20–25% of pregnant women report drinking alcohol at some time throughout pregnancy [3,4]. Further, the present health recommendation message in France stating “zero alcohol” consumption during pregnancy is gaining momentum as an issue because many studies indicate that low to moderate ethanol exposure during pregnancy may also produce enduring deficits in the adult including learning and memory dysfunction. In this context, it is important to better understand the effects of prenatal alcohol exposure on cellular mechanisms of learning and memory. 

The large variety of disorders induced by early-life ethanol exposure is encompassed under the term fetal alcohol spectrum disorder (FASD). FASD affects about 1% of living births according to the World Health Organization and this makes alcohol exposure during pregnancy the leading cause of irreversible mental retardation although it remains preventable. FASD includes cases of all levels of prenatal ethanol exposure, from heavy to light or moderate exposure during gestation. The most dramatic case is fetal alcohol syndrome (FAS), first medically acknowledged and reported by Dr Lemoine, a French pediatric, in 1968 (in Les enfants de parents alcooliques: anomalies observées à propos de 127 cas. Translated as “Outcome of children of alcoholic mothers: anomalies observed in 127 cases”. Ouest Med. 1968; 8, 476–482) before being fully and scientifically described in the early 1970s [5]. In particular, these last authors listed FAS-typical craniofacial abnormalities accompanied by growth retardation and CNS dysfunctions including irreversible impairments in learning and memory. 

Learning and memory within the CNS involve several brain regions; however, preclinical and clinical studies have revealed an important role for the hippocampus not only in the acquisition of episodic memories, but also in the consolidation and retrieval of these memories [6]. The hippocampal formation has attracted much attention since its role in human memory was reported in the 1950s with the patient Henri Molaison (1926–2008). Subsequently, electrical recordings of the hippocampus in animal models revealed the specific physiology of this structure in the early 1970s [7,8], although many other researchers had long been analyzing the nature of memory in humans [9]. Nevertheless, as observed in Henry Molaison, lesions of the hippocampus produce anterograde amnesia (i.e., incapacity to store new memories) while older memories are keep intact. Furthermore, the neuronal network of the hippocampus revealed a specific electrophysiological property called synaptic plasticity that was recorded for the first time by Bliss and Lomo in 1973 in anaesthetized rabbits. Alcohol (ethanol (EtOH)) is known to disturb memory at any age throughout human life via alterations of the hippocampus neuronal network physiology [10]. The present chapter addresses the effects of ethanol on synaptic plasticity in the hippocampus after early-life ethanol exposure during pregnancy (i.e., prenatal ethanol exposure (PEE)).

## 2. The Hippocampus and Long-Term Depression of Synaptic Plasticity

Because of his intractable epileptic conditions, Henry Molaison underwent surgery consisting of bilateral resection of the medial temporal lobe. This intervention revealed that the hippocampus, a bilateral structure located underneath the cortex of the medial temporal lobe, has an important role in the physiology of human memory. Later, the hippocampus was linked to the brain limbic circuit. At the macroscopic level, the hippocampus has a more or less horseshoe shape in mammals, but because of its inner winding of cellular areas defining its ultrastructure organization, it resembles a seahorse from which it finally holds its scientific name. Indeed, the word “hippocampus” is from the Greek “hippo” meaning “horse” and from “kampos” meaning “sea.” The neuronal ultrastructure of the hippocampus is almost identical in all mammals and demonstrates strong physiological connections to other nearby structures including the dentate gyrus, the pre- and parasubiculum, and the entorhinal cortex. Together, this relative large ensemble is called the “hippocampal formation.” At the neuronal network level, the hippocampus is composed of three main areas: the dentate gyrus (DG), the Cornu Ammonis field (CA, named after Amun, a god from the ancient Egypt) and the subiculum. These internal subdivisions of the hippocampus are interconnected through a serial and unidirectional tri-synaptic pathway. Physiologically, the major input to the hippocampus enters via the entorhinal cortex to reach the granule cells of the DG via the perforant axonal path. Axons from the granule cells project then to the CA3 area, one of the CA field’s subdivisions, via the mossy fiber pathway. Each of these CA subdivisions is composed of pyramidal cells as principal cells that are connected to one another. From CA3, the axon’s collaterals form the Schaefer’s collateral path to reach CA1 pyramidal cells. From this last area, the signal reaches the subiculum, which sends a major input back to the entorhinal cortex/parahippocampal region [11]. Finally, the projections from the parahippocampus return to the neocortical association areas from which the inputs to the medial temporal lobe originate. Importantly, the synapses connecting the principal cells along the tri-synaptic pathway within the hippocampus are excitatory and use glutamate as the neurotransmitter.

Because the hippocampal formation participates in the formation of memories, including the consolidation of long-term memory in both human and animals, the physiology of this structure should be able to sustain such an important role. Specifically, this structure should present a high degree of both functional and structural plasticity, providing the brain with dynamic performances. This particular property would allow for flexibility and proper regulation of behaviors according to the environment and experiences. Functional synaptic plasticity is a property that either increases or decreases synaptic strength in response to a previous history of stimulation. This property is found at each of the glutamatergic synapses of the hippocampal tri-synaptic pathway and is basically bidirectional: If a persistent decrease of synaptic strength below baseline value measured before the induction of such a decrease is obtained, then it is referred to as long-term depression (LTD) of synaptic activity. On the contrary, if an increase of synaptic strength is obtained, it is called long-term potentiation (LTP), the functional opposite of LTD. Furthermore, functional synaptic plasticity, in the form of either LTP or LTD, changes across time once it is induced. Therefore, we distinguish short-term and long-term plasticity. Short-term plasticity lasts milliseconds to several minutes, and long-term lasts several hours to days or more. These changes affect the functionality of the synapse and occur according to the previous history of stimulation of the synapses in the hippocampus, and are considered today as the main cellular events that encode and consolidate new memories. Thus, adjustment of synaptic strength is a necessary property to acquire new hippocampus-dependent learning [12,13]. Distinct cellular mechanisms underlie the induction and the maintenance phases of bidirectional synaptic plasticity. These mechanisms can be located at pre- or postsynaptic sites and they can also differ depending on the duration of the induced plasticity: short- versus long-term plasticity. *N*-methyl-d-Aspartate (NMDA)-dependent forms of LTD and LTP in the CA1 area have been reported, as well as metabotropic glutamate receptor (mGluR)-dependent forms in both the CA1 and DG areas [14]. Other forms of LTD in these two areas involve the endocannabinoid system [15,16] or postsynaptic voltage-dependent calcium channels [17]. In this review, I will focus on the long-lasting form of NMDA-dependent LTD since it is this form that has been most studied in the context of PEE.

Concerning LTD, our research group has focused on the NMDA-dependent form recorded in the CA1 area. Typically, this form of LTD is experimentally induced through the stimulation of Schaeffer’s collaterals using 600 to 900 square electrical pulses applied at low frequencies between 1 and 5 Hz. Some other electrical stimulation paradigms consist of low-frequency paired pulses separated by 200 ms. Both procedures of LTD induction probably lead to less calcium influx into the postsynaptic cell through the NMDA receptors as compared to LTP induction. Consequently, this “low” amount of free calcium entering the cell triggers protein phosphatases, which in turn dephosphorylate AMPA (α-amino-3-hydroxy-5-methyl-4-isoxazolepropionic acid) receptors of the postsynaptic membrane leading to their removal and internalization. As a result, the response of the synapse to the same stimulus tested before induction of LTD becomes smaller and the synapse is less prone to transfer action potentials throughout the network. This LTD can be blocked in the presence of an antagonist of NMDA receptors. As noted above, another form of LTD in CA1 depends on the activation of mGluRs [14]. In vitro, on acute hippocampus slices, mGluR-dependent LTD can be triggered chemically with bath application of DHPG, a group I mGluR agonist [18,19]. This type of LTD can also be induced electrically in vitro with a paired-pulse protocol applied at low frequency [20], whereas in vivo (i.e., in freely moving rodents), the mechanism appears more complicated [21]. As for NMDA-dependent LTD, the cellular mechanisms triggering mGlu-LTD in the hippocampus may involve both pre- and post-synaptic events. Among the glutamatergic receptors, for example, mGluR5 is well identified as the post-synaptic target both in vivo [22], where it works in association with the NMDA receptor, and in vitro [19,23]. Although the intimate cellular events involved in both electrically and chemically induced LTD may differ [14], both are maintained in the long term because of internalization of AMPA receptors from the post-synaptic membrane [24]. Finally, further differences may exist between NMDA-LTD and mGluR-LTD in the hippocampus, as it has been shown that both types of LTD involve differing protein kinases and phosphatases, as well as distinct changes in tyrosine phosphorylation [14]. However, neither endocannabinoid-dependent LTD nor mGlu-LTD have been tested after PEE. 

## 3. Hippocampus Long-Term Depression after PEE

Irreversible ethanol-induced deficits in cognitive function are well described and these deficits include learning and memory impairments, which often reflect damage in the hippocampus [25]. Regarding developmental ethanol exposure, experiments in rodent offspring, and in humans, have shown a reduction in spatial learning capabilities after PEE [26,27,28,29]. Specifically, in 7-day-old rats, a binge-like EtOH treatment consisting of 2 acute i.p. doses of 2.5 g/kg ethanol given 2 h apart (blood ethanol content, BEC 500 mg/dL, then 200 mg/dL > 10 h) impaired spontaneous alternation in a Y-maze three weeks later [30], but did not impair water maze learning or retrieval, although others [31] have reported deficits in this last test. In addition, LTD was abolished in the CA1 area of hippocampal slices obtained from 30-day-old animals [32].

Other information is available from longer exposure to EtOH, either throughout the entire gestation period or the entire period of brain development in rodents, including pre- and postnatal exposure. Such conditions model human conditions under which pregnant women with alcohol use disorder cannot abstain from alcohol consumption for the entire gestation period. One study revealed that EtOH exposure throughout gestation in rats (BEC ca. 192 mg/dL at gestational day 15) did not affect CA1-LTD induction in vivo in postnatal day (PND) 30–35 male offspring, but reduced LTD in females [33]. Interestingly, this study revealed the sexual dimorphic effects of PEE on LTD. In another study [34], rats were exposed to EtOH pre- and postnatally, covering the entire brain developmental periods in rodents, modeling the equivalent of the three trimesters of human gestation. With such exposure, BEC in dams was measured at about 100 mg/dL during lactation. Electrophysiology was done in the CA1 area of late-adolescent male rat offspring (PND45–55) using acute hippocampus slices. In this condition, it was found that LTD was enhanced, which was confirmed because an inefficient electrical stimulation in both control and pair-fed animals was able to induce LTD after EtOH exposure. Furthermore, this form of enhanced LTD was selectively blocked with an antagonist of the GluN2B subunit of the NMDA receptor. Taken together, these two last studies suggest that exposure to EtOH during the three-trimester equivalent of human gestation is necessary to increase LTD in male offspring, but understanding the results obtained in female offspring requires further experiments. Moreover, the two studies differed in the approaches used by the respective authors (in vivo or ex vivo). Nevertheless, to the best of our knowledge, these two studies are the only ones that have investigated LTD in the hippocampus after PEE, as others [35,36] have studied depotentiation of the synapse rather than LTD. Indeed, depotentiation, the reversal of induced LTP, is mechanically different from LTD induced from a basal network activity level [37] and can still be observed while LTD is abolished [37]. Specifically, both homosynaptic LTD and depotentiation rely on postsynaptic AMPA receptor endocytosis, but the mechanisms implicate differential dephosphorylation processes of the GluA1 subunit of the AMPA receptor. Interestingly, while CA1-LTD can be triggered either through the activation of NMDA or mGluRs, it has been shown that activation of both types of receptors are required to trigger depotentiation at CA3-CA1 synapses in vivo [38,39,40]. Despite mechanical differences between LTD and depotentiation, some mechanisms involved in both signals may be affected after PEE. Nevertheless, the work from An and colleagues suggest an interesting imbalance between LTD and LTP after PEE since they found sexually dimorphic effects of EtOH on depotentiation after intragastric gavage of EtOH (4 g/kg/day, from 7 days before mating until delivery). Specifically, depotentiation was enhanced in males, but absent in females [35,36,41].

## 4. Glutamate Neurotransmission, LTD, and Prenatal Ethanol in the Hippocampus

At the cellular level, EtOH-induced cognitive deficits have been attributed to an activity-dependent impairment in synaptic plasticity in the hippocampus [42,43,44,45,46,47]. Learning and memory deficits initially implicated a reduction in NMDA-dependent synaptic long-term potentiation (LTP) [43,44]. However, recent results have also revealed a change in the LTD signal in the same CA1 area, with LTD being either increased in offspring after PEE [34] or decreased [33]. As a consequence, learning and memory processes that involve LTD specifically should be altered after PEE, and, if LTD is involved concomitantly to LTP in some types of learning, these also should be altered. In other words, we must take into account not only that LTP is reduced after PEE but also that LTD is altered and that learning and memory deficits after PEE probably involve changes in both of these cellular phenomena either spontaneously or in specific learning tasks. Unfortunately, there is a paucity of studies regarding the fate of LTD after PEE, and there is an even greater lack of proposed mechanisms to explain PEE-induced LTD alteration. One putative mechanism for aberrant LTD enhancement is a synaptic reorganization of the NMDA receptor subunits [34]. These authors found that GluN2B subunit of the NMDA receptor was overexpressed in the synaptic compartment (and not extrasynaptically) and that ifenprodil, an antagonist of GluN2B subunit, selectively blocked aberrant LTD. They also demonstrated, although indirectly with pharmacological tools, that aberrant LTD was of synaptic origin. Globally, it has been proposed that, after PEE, the synapse accumulates GluN2B-containing NMDA receptors that facilitate LTD induction. Given the age of the animals in which this study was performed, such configuration of the synapse resembles that of immature tissue. Indeed, in adult animals, GluN2B-containing receptors predominate at peri- and extrasynaptic sites [48,49]. Moreover, and although it is certainly not as simple as that, GluN2B is involved in LTD induction according to many pharmacological experiments performed in different brain areas [50,51]. Altogether, PEE may delay maturation of excitatory synapses with respect to both receptor structure and function. 

The electrophysiology properties of the NMDA receptor depend on its composition in subunits. Notably, the amount of free calcium flowing through this receptor is larger in GluN2B- containing NMDA receptors compared to GluN2A-containing ones [50], and because bidirectional synaptic plasticity is at least partly determined by the postsynaptic calcium concentration, it is important to measure the effects of PEE on NMDA-receptor subunit expression. The NMDA receptor is a heterotetramer complex that includes three different subtypes of subunits, i.e., GluN1, GluN2, and GluN3. NMDA receptors are composed of two GluN1 subunits that ensure the function of the receptor and are associated with two regulatory subunits from any combination of GluN2 and/or GluN3 subunits. In particular, four isoforms of the GluN2 subunit (from A to D) determine the final NMDA receptor electrophysiological properties [50]. According to the literature, GluN1 subunit expression and its association with (postsynaptic density95) PSD95 are probably not changed in the hippocampus or other brain areas after PEE [52,53]; however, some authors reported an increase in GluN1-subunit expression in adult brain after PEE [54]. This result includes some of the splice variants [54] and others have reported an increase in the dentate gyrus for the C2’-containing GluN1 NMDA receptor [52]. GluN2A subunit expression was reduced after PEE in the forebrain at PND14 and its cell surface expression was also decreased at PND21 [54,55]; however, GluN2A was increased in the hippocampus at PND 10 after pre- and postnatal exposure up to PND9 [52]. A decrease in GluN2A expression in hippocampal homogenates also was reported with no changes in GluN2A-PSD95 association [56]. GluN2B subunit expression was decreased in the forebrain at PND14 but unchanged at PND21 [54,55], although GluN2B was increased in some subregions of the prefrontal cortex, an effect corroborated with pharmacological tests [57]. GluN2B was decreased in the hippocampus at least at PND7 in rats or in adult mice detected as a decrease in PSD95-GluN2B complex association [56], but others did not report similar results [54]. GluN2B was reduced [58] in the DG of the hippocampus, while some authors [34] found it to be increased in the CA1 area. This last finding was confirmed with pharmacological tests in the cited study. Interestingly, learning deficits in adult rats after pre- and postnatal EtOH exposure was accompanied by dysregulation in hippocampal gene expression through a significant induction of glutamate-related genes including those for GluN2A to D subunits [59]. Overall, it is difficult to determine the definitive picture of PEE effects on the expression levels of the NMDA receptor subunits. Nonetheless, some studies clearly suggest that differences in subunit expression can be rather opposite within the same structure if the measurements are performed in the different subfields of the brain area being studied. The example of divergent results in GluN2B expression between the hippocampus DG and CA1 areas is particularly intriguing [34,58]. In the same vein, this difference also seems to exist for GluN2A in the forebrain and prefrontal subfields [53,54,55]. In addition to this level of analysis, it must be noted that each of the studies used different paradigms of ethanol exposure, different animal species, and even different substrains of rat. Therefore, although LTD in the hippocampus is altered with changes in NMDA receptor function after PEE, the possible link between these results and the expression level of NMDA receptor subunits is not yet clearly established.

mGluRs, in addition to NMDA receptors, are also modified after PEE. The expression of mGlu5 in the DG of adult offspring is decreased after PEE with a possible presynaptic site of action [60]. After PEE, the application of trans-ACPD, a selective agonist active at both Group I and Group II mGluRs (which include mGlu2, mGlu1, mGlu5, and mGlu4 receptors), transiently enhanced PIP2 hydrolysis for two weeks in 14-day-old pups [61]. Others found that adult offspring from dams fed an ethanol solution of 5% or greater throughout gestation produced a reduction in trans-ACPD stimulated IP1 accumulation at 10–13 days of life [62]. Another effect of PEE is the modulation of AMPA-Rs signaling. Indeed, PEE to a 3.35% ethanol solution throughout gestation did not alter the agonist concentration–response curve for AMPA-induced depolarization in CA1 pyramidal neurons [62], whereas consumption of a 5%-ethanol liquid diet during the same period was shown to decrease synaptosomal GluA1 receptor subunit expression in the DG of female adult offspring without producing changes in GluA2 subunit expression [63]. Interestingly, in this latter study, cytoplasmic activity-regulated cytoskeletal (ARC) protein levels were increased after PEE. Given the role of ARC in regulating synaptosomal AMPA-Rs, these results suggest a reduction in activity-dependent changes in AMPA-Rs in a way that diminishes the involvement of AMPA-Rs in DG synaptic plasticity. Specifically, Group I mGluRs-LTD is accompanied by AMPA-Rs endocytosis [64] and increased dendritic synthesis of ARC, a component of AMPA-Rs endocytosis [65]. Similarly, pregnant rats that received ethanol via gavage (4 g/kg/day, 38% *v/v*) throughout gestation showed impairment in CA1 AMPA-Rs-mediated neurotransmission (i.e., decreased miniature excitatory postsynaptic currents; mEPSCs) that was interestingly corrected after early postnatal or adolescent sub-chronic treatment with aniracetam, a nootropic compound and an allosteric modulator of AMPA-Rs [66,67,68].

## 5. GABAergic Neurotransmission, LTD, and Prenatal Ethanol in the Hippocampus

In contrast to other psychoactive substances, ethanol does not require a specific type of membrane receptor to be effective in the CNS, but the GABA_A_ receptor is another important target for ethanol during brain development. Interestingly, it has been shown that 3 mM ethanol modulates the activity of the GABA_A_ receptor [69], and this concentration corresponds to one standard alcoholic beverage. Indeed, ethanol is a positive allosteric modulator of the GABA_A_ receptor, increasing the activity of the receptor without binding at the endogenous ligand site. Furthermore, GABA_A_ inhibition participates in the modulation of LTD and LTP by restraining expression of both signals in mature naive animals [70,71]. After PEE, an increased sensitivity of the GABA_A_ receptor to its positive allosteric modulators has been reported in 5-month-old rat hippocampus, while the effects of negative allosteric modulators were decreased [72]. Such changes in sensitivity to allosteric modulators may come from different levels of receptor expression. For example, an increase in the expression of some subunits of the GABA_A_ receptor (i.e., β2/3, α1, and α5) in the hippocampus of young-adult guinea pig offspring and a decrease in adult mice brains has been reported [73,74,75,76]. However, PEE did not change the binding for several GABA_A_-related substances ([^3^H]flunitrazepam, [^3^H]TBPS, and [^3^H]muscimol) in the hippocampus [34], nor did it change the *Bmax* value for [^3^H]muscimol in subfields of the hippocampus [74,75], suggesting that the binding capacity of the GABA_A_ receptor was not affected by PEE despite possible changes in subunit composition of the receptor. Thus, disturbances in GABA inhibition after PEE may come from a dysfunction of the GABA_A_ receptor. The GABA_A_ receptor is an ionotropic receptor allowing a chloride influx into the cell to hyperpolarize the membrane. PEE did not alter GABA-stimulated chloride efflux measured from membrane vesicles in adult offspring nor electrically evoked glutamate or GABA release from hippocampal slices in baseline conditions [77], but activity-dependent potentiation of GABA release after a tetanizing stimulation was in fact increased after PEE, an effect that could mediate the modulation of LTP amplitude after PEE. The basal chloride influx during GABA action is due to the concentration gradient of Cl^−^ between the inside and the outside of the cell, a gradient produced by the activity of NKCC1 (Na^+^-K^+^-Cl^−^ co-transporter-1) and KCC2 (K^+^-Cl^−^ co-transporter-2), a chloride importer and a chloride exporter, respectively. Interestingly, during brain development, GABA_A_ receptors are excitatory as a consequence of high intracellular [Cl^−^] in immature neurons because they predominantly express NKCC1 [78]. During maturation, an increase in expression of KCC2 allows the functional switch of GABA_A_ receptors to inhibitory effects [78]. However, ethanol exposure in rat during the equivalent human third trimester of gestation did not change chloride co-transporters expression in the CA3 area of neonatal rat hippocampus slices [79]. Further, the developmental profile of giant depolarizing potentials, which depend on the excitatory effects of GABA_A_ early in life, was also unaltered [80] and acute ethanol did not affect either mRNA and protein expression of NKCC1 and KCC2 in neocortical slices [81]. Interestingly, in contrast to these studies, a selective overexpression of the functional isoform of KCC2 in the CA1 area was recently reported after PEE during the three-trimester equivalent of human gestation, [82]. In addition, this functional isoform was probably inserted into the pyramidal cell membrane. In turn, this effect may have increased the concentration gradient of chloride in neurons after PEE, leading to stronger GABA_A_-mediated inhibitions within the network once the channel is activated. However, this assumption still lacks the direct scientific evidence of a change in the reversal potential for GABA_A_ inhibitions in CA1 pyramidal cells after PEE. Although these results may conflict with some of the studies reviewed above, ethanol exposure, time of exposure, and specific subfield effects of PEE may partially explain differences in the findings.

## 6. Physiological Role of LTD and the Possibility of Restoring It after PEE

Although the role of hippocampal LTD is still poorly understood at the behavioral level, there is growing evidence suggesting a role for this form of plasticity in novelty acquisition since LTD induction is facilitated by novelty [83,84]. Behavioral flexibility, spatial working memory, and, more precisely, consolidation of long-term spatial memory [85,86,87] are other possible roles for LTD. For example, learning deficits in object recognition occurred after a viral transfection that selectively abolished LTD [88] and spatial working memory is altered in mice lacking calcineurin, a calcium-dependent protein phosphatase, while LTD is abolished in these mice [89]. Interestingly, enhancement of CA1-LTD after an acute stress impaired spatial memory retrieval [90] and a facilitated LTD in this same pathway enhanced contextual fear memory extinction [91]. Others found faster learning in spatial object location and an increase in fear memory in the presence of an enhanced LTD [92]. Altogether, these studies suggest that enhanced LTD after PEE as seen in male offspring [34] or reduced LTD as reported by others [32,33] should impact at least some learning capabilities. Therefore, restoring LTD is as important as rescuing its functional opposite, LTP. Among the different strategies known to correct some of the effects of fetal alcohol syndrome at behavioral or cellular levels, only a few have been tested in regard to synaptic plasticity in the hippocampus [93]. In addition, most of the attempts to correct synaptic plasticity deficits after PEE have been aimed at restoring LTP. Exercising for a few days restored DG-LTP in male rats after PEE [94] with better performance in the Morris water maze. Postnatal nutritional supplementation with *N*-acetylcysteine (N-Ac) from Days 23 to 60, and omega-3 fatty acids from birth to adulthood have been also shown to ameliorate DG-LTP in male rats after PEE [95]. N-Ac had no effects in female offspring who did not show alteration in DG-LTP after PEE, however. Interestingly, the combination of both exercise and omega-3 fatty acid supplementation possibly act in synergy to improve cognition [96]. N-Ac is the precursor of glutathione (GSH) synthesis in the brain and possibly corrects LTP through its property as a redox modulator. Similarly, omega-3 fatty acids also show some antioxidant properties and PEE is known to decrease some of these fatty acids in the brain leading to disturbances of signaling pathways that may ultimately affect synaptic plasticity. Prenatal choline supplementation also seems beneficial for LTP [97]. Reduced in vitro LTP seen after PEE was also corrected by blocking histamine H3 receptors in vivo 2 h before electrophysiology experiments [98]. In mice, an injection of chloride-lithium 15 min before an acute injection of ethanol at PND7 prevented ethanol-induced neuronal death and rescued LTP in adults [99]. In a recent study [82], in vitro CA1-LTP was rescued after PEE in young adult male offspring using bath application of bumetanide, a diuretic and a dose-dependent blocker of chloride-potassium co-transporters NKCC1 and KCC2 [100]. As reported here, all studies addressed LTP and we do not know whether LTD can also be corrected with the same already tested factors. Nonetheless, bumetanide applied in vitro was also able to rescue both LTP and LTD in acute hippocampal slices of male offspring after PEE [82]. Finally, only one study found a way to correct both and concomitantly LTP and LTD disturbances in male offspring after PEE [82]. This experiment further supports the assumption that PEE-induced chloride homeostasis disturbances through modulation of KCC2 probably affects GABA_A_ inhibitions and ultimately bidirectional synaptic plasticity in CA1 area. In addition, this study is the first one showing that a single pharmacological agent is able to correct both synaptic signals at the level of the hippocampus after PEE. Importantly, an attempt for an in vivo treatment with bumetanide has been performed (Pierrefiche et al.; unpublished data) and revealed a partial recovery of LTD in slices of PEE-exposed animals. Although this in vivo attempt to correct both LTP and LTD simultaneously was encouraging, it must be kept in mind that humans with FAS are prone to present seizure activity and that manipulating the GABAergic system could be extremely complicated in such cases. Nevertheless and interestingly, GABA inhibitions have been proposed for some years as a major target in some neurodevelopmental psychiatric diseases such as autism or fragile X syndrome, in which hippocampus LTD is greatly disturbed. Bumetanide is effective in correcting some of the features of these two neurodevelopmental disorders in both animal models and humans [101,102]; however, in these latter studies, KCC1 was the target to be corrected and not KCC2. Another limitation is that bumetanide poorly crosses the blood–brain barrier [100]. Therefore, in vivo treatment with bumetanide after PEE in rats needs to be repeated with adjustments to the dose and possibly the additional use of probenecid, a substance that increases blood–brain permeability to bumetanide [103]. 

## 7. Concluding Remarks

Despite all efforts to better understand the mode of action of EtOH during brain development and in particular its effects on LTD and LTP, it is acknowledged that ethanol-induced damages during gestation also depend upon several factors that are difficult to control in the human population. The exact dosage of ethanol is rarely known, and the timing and frequency of exposure remain difficult to estimate. The genetics and metabolism of the mother are two additional factors to take into account. Nevertheless, taken together, the studies presented herein all indicate that PEE effects on hippocampal synaptic plasticity involve both glutamatergic and GABAergic neurotransmissions concomitantly and that cognitive deficits after PEE arise from disturbances of the two main types of synaptic plasticity signals underlying learning and memory. However, because a definitive picture of the effects of prenatal EtOH on the cellular mechanisms of learning and memory is not yet available, further investigations on the mechanisms of action of ethanol are warranted.
